# Bibliometric visualisation of industrial and organisational psychology during COVID-19 pandemic: Insight for future research

**DOI:** 10.4102/sajip.v48i0.2007

**Published:** 2022-09-28

**Authors:** Ufi Fatuhrahmah, Herlina Siwi Widiana

**Affiliations:** 1 Department of Psychology, Faculty of Psychology, Universitas Ahmad Dahlan, Yogyakarta, Indonesia

**Keywords:** industrial-organisational psychology, bibliometric, COVID-19, pandemic, mapping

## Abstract

**Orientation:**

Industrial and organisational psychology (IOP) researchers have shown their contribution to solving COVID-19 pandemic in the workplace through the enormous number of studies.

**Research purpose:**

This study intended to map IOP research related to the COVID-19 crisis to provide the research issues that have emerged and potential for future research.

**Motivation for the study:**

All the IOP levels (worker, team and organisation) were impacted by COVID-19, and they continuously change. Researchers must be careful in directing their research and avoid focusing on certain levels or problems.

**Research approach/design and method:**

A bibliometric visualisation analysis method was adopted in this study.

**Main findings:**

The bibliometric results showed that the prominent keywords in IOP research-related COVID-19 are ‘human(s)’, ‘COVID-19’, keywords related to subject characteristics and mental health. Six clusters on the map showed the prominent themes: mental health, health care workers as the research subject, specific workplace issues, digital technology, methodologies used, and country. Furthermore, in every cluster, the depth overview of study results is presented. The top issues were at the worker-level, while the organisational-level issues gained limited attention.

**Practical/managerial implications:**

For practitioners and managers, this study provides a complete picture of emerging issues during COVID-19 crisis ranging from causes, risk factors and solutions. For researchers, this study can provide insight for further research.

**Contribution/value-add:**

This study provides a comprehensive overview of the IOP issues related-COVID-19 that will be beneficial as the basis for policymaking and recommendations for future potential areas.

## Introduction

The World Health Organization (WHO) has declared the spread of coronavirus disease 2019 (COVID-19) to be a global pandemic since 11 March 2020. Researchers worldwide are working hard to solve the problems in their respective fields. The WHO research database shows that 174 676 articles related to COVID-19 covered many disciplines, mainly in the clinical aspects of COVID-19 (World Health Organization, 2020). A bibliometric analysis conducted using the Scopus database in April 2020 shows that researchers have produced 3513 scientific papers, with the largest presentation of research in public health (Hamidah, Sriyono, & Hudha, 2020). China and the United States of America (USA) have become the centre of COVID-19 research (Hamidah et al., 2020). Specifically in psychology, several bibliometric analyses showed the USA was the most prominent country that published impact research (Dong et al., 2022; Ho, Fu, & McKay, 2021), the majority are in clinical psychology and psychopathology (Ho et al., 2021) and more focused on mental health (Dong et al., 2022; Ho et al., 2021; Zambrano, Alvarez, & Caballero, 2021). However, we have not found any research focused on industrial and organisational psychology (IOP).

Coronavirus disease 2019 impacts not only health and clinical aspects but economic growth, jobs and welfare as well (Olivia, Gibson, & Nasrudin, 2020). As the longest pandemic in the 21st century, the COVID-19 pandemic caused the worst global recession since the Second World War (World Bank, 2020). The World Bank predicts that the effects of this pandemic will last in the long term (10 years ahead) and will primarily impact economic growth worldwide (World Bank, 2020). Considering the phenomenon and projection, IOP must play a significant role for several reasons. Firstly, IOP has a broad scope of knowledge at the individual, group and organisational levels (American Psychology Association, 2008). Studies and research at various levels in IOP are crucial to formulating comprehensive solutions during and after the COVID-19 pandemic. Secondly, IOP is probably more relevant than ever to work lives, organisations and society (Ones, Kaiser, Chamorro-Premuzic, & Svensson, 2017). Further, IOP is expected to play a role both in the recovery process and in anticipation of the negative impacts that will occur in the workplace. Therefore, it is essential specifically to map and review IOP studies during the COVID-19 pandemic.

The pandemic has accelerated the pace of change within organisations, which is likely to change post-pandemic working conditions, new practices, policies and systems (Malhotra, 2021). The experts argued that several areas were changed and needed attention during the COVID-19 pandemic at various levels (Kaushik, 2020; Kniffin et al., 2021; Rudolph et al., 2021): firstly, changes in worker level, for example, safety and health, work–family issues, job insecurity, mental health and well-being, as well as skill and competencies requirements; secondly, changes at the group level, including virtual teams and team leadership; and lastly, changes at the organisational level, which include virtual work, precarious work, human resources, the aging workforce, adoption of technology and people connection. Kniffin et al. (2021) highlight the moderating factors that distinguish the impact of the COVID-19 pandemic to organisation, including individual characteristics and organisational norms.

However, in IOP, these changes and practices are encouraged to be evidence-based, with some of this evidence coming from research (Bartlett & Francis-Smythe, 2016). Numerous arguments from the IOP experts may raise the question of whether the IOP researchers have addressed all emerging issues instead of focusing on a certain level and themes. Hence, this study intends to map the IOP research trend using bibliometric analysis for two purposes. Firstly, the bibliometric analysis will provide a map of prominent themes and their coverage that has been focused on during the COVID-19 pandemic. Secondly, the current research map gives insight about potential future studies that are essential but have not been covered.

The use of bibliometric analysis has grown rapidly because of its ability to analyse large bibliometric networks, ranging from citation relationships, co-authorship relationships and co-occurrence relationships between keywords used in publications (Van Eck & Waltman, 2014). During the COVID-19 pandemic, there were three most-cited bibliometric studies in the psychology subject area, presenting various networks from citation, co-authorship, country, and co-occurrence of keywords (Dong et al., 2022; Ho et al., 2021; Zambrano et al., 2021). However, to discover up-and-coming fields and untapped potential fields of research, it is recommended to use content analysis through harvesting keywords (Ellegaard & Wallin, 2015; de Olieveira et al., 2019) and focusing on co-occurring keywords extracted from titles, abstracts or even full-text analysis (Song & Chambers, 2014). Therefore, the study only focuses on the co-occurrence of keywords in the IOP area and deepens the analysis by resuming the research results. It is beneficial to capture the current issues that are covered and thus utilise them as the basis for future research or policies both in pandemic and post-pandemic periods.

## Method

This study applied bibliometric analysis with VOS viewer software. Bibliometric analysis is the method to map the state of the art of scientific themes for various purposes, such as identifying research gaps and trends through keyword analysis, analysing scientific performance (articles, authors, institutions and journals) and clustering scientific gaps from publications (de Olieveira et al., 2019). There are currently many programmes available to run this analysis, each with its advantages, such as BibExcel, VOSViewer, CiteSpace, Gephi and Pajek.

**FIGURE 1:**
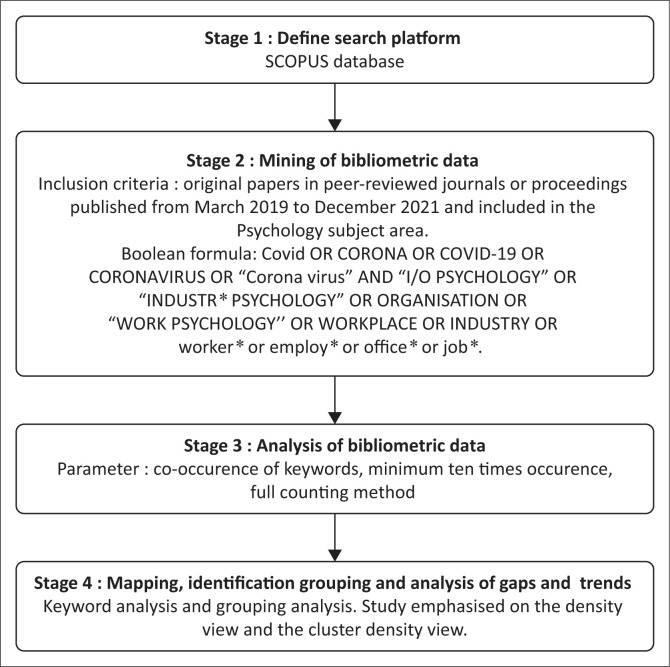
Stages for bibliometric analysis.

The study used VOSViewer because it allows text mining and provides low-dimensional visualisation in which the distance reflects their similarity as accurately as possible (Van Eck & Waltman, 2007), which aligns with the study purpose. VOSViewer is free software that is useful for displaying large bibliometric maps in four different views, namely the label view, the density view, the cluster density view and the scatter view (Van Eck & Waltman, 2010). The study applied four steps in bibliometric mapping, as shown in Figure 1.

Specifically, in stage 4, the analysis emphasises the density view and the cluster density view because they indicate the most critical areas, the themes’ density and the relationship between the themes. In the density view, items in the center of the map with bigger and bold labels are indicated as the most prominent and most discussed areas on a map. The cluster density view shows the grouping and the relationship between the themes. Depth analysis from the cluster density view was performed for comprehensive information about the themes, ranging from the causes, risk factors and solutions that the researcher has studied during the COVID-19 pandemic.

### Ethical considerations

This article followed all ethical standards for research without direct contact with human or animal subjects.

## Results

**FIGURE 2:**
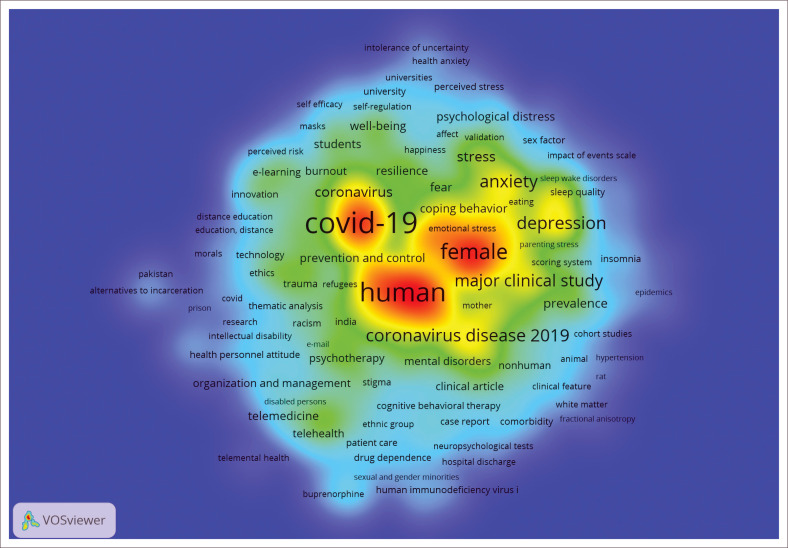
The density visualisation.

The Boolean formula search resulted in 5399 articles from the Scopus database. Data analysis through VOS Viewer 1.6.15.x resulted in 16 934 keywords, of which 935 met the threshold. Based on the density view on a map, ‘Human(s)’ is the most used keyword in research studies, with a total of 3105 co-occurrences and a total link strength of 62 938, as shown in Figure 2.

The detail prominent keywords are presented in Table 1, including occurrences and total link strength. The top 15 keywords showed that keywords ‘human(s)’, ‘pandemic’, ‘mental health’ and keyword-related subject characteristics dominate IOP studies during the COVID-19 pandemic.

**TABLE 1:** The most prominent keywords.

Keyword	Occurrences	Total link strength
Human	1752	34 182
Humans	1353	28 756
COVID-19	2451	25 783
Female	1024	23 751
Article	1049	23 296
Adult	979	22 861
Male	976	22 751
Pandemic	1054	19 993
Coronavirus disease 2019	574	16 150
Pandemics	631	14 767
Psychology	582	14 111
Major clinical study	439	11 983
Anxiety	581	10 671
Mental health	561	10 127
Depression	535	10 105

**FIGURE 3:**
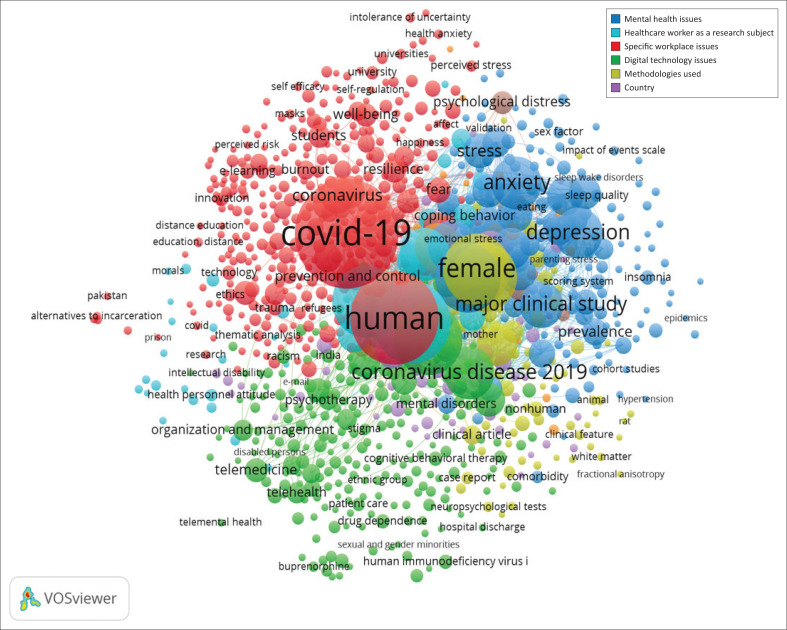
The cluster density visualisation.

The cluster density view (Figure 3) shows eight clusters, but two clusters were too small with fewer than 10 keywords and consisted of similar keywords in another cluster. Furthermore, two clusters were eliminated from the analysis.

Because of the elimination of two clusters, six clusters were formed, namely mental health issues, health care workers as a research subject, specific workplace issues, digital technology issues, methodology and country. Table 2 shows clusters and keywords in each cluster (order based on the frequency of occurrence).

**TABLE 2:** The clusters and keywords.

Cluster	Keywords
Mental health issues	Anxiety, depression, stress disorder, occupational stress, mental health, mental disorder, psychological distress, mental stress, post-traumatic stress disorder, somatisation, mood disorder, insomnia
Health care worker as a research subject	Health care workers, health personnel, nurses, personnel hospital, physicians, health care professionals, nursing staff, health care personnel, health practitioner.
Specific workplace issues	Burnout, job or occupational stress, fatigue, workload, job insecurity, work from home or remote work, leadership, job satisfaction, work engagement, work or task performance, entrepreneurship, work–life balance, productivity
Digital technology issues	Telemedicine, artificial intelligence, teleconsultation, telecommuting, teleworking, e-learning, tele-rehabilitation, machine learning, tele-mental health, data analytics, digital transformation, virtual teams
Methodologies used	Cross-sectional study, survey and questionnaire, longitudinal studies, qualitative research
Country	United States, China, Italy, India, Spain, Iran, Japan, Israel, Pakistan

*Source:* The original stages by de Olieveira et al (2019)

## Discussion

### Outline of the results

The bibliometric visualisation shows that keywords ‘human(s)’ and ‘COVID-19’ are the two highest occurring keywords. As the main subject in IOP science, it is not surprising that ‘human(s)’ rises as the prominent keyword in IOP studies during the COVID-19 pandemic. Besides the human(s) keyword, the study found that the research subjects who were primarily involved in IOP studies are female, male and adult. This finding showed the position of IOP’s study focus on people at work (Levy, 2017), characterised by the gender and adult age. Furthermore, as resulted in other bibliometric studies, either in psychology in general (Zambrano et al., 2021) or clinical psychology (Ho et al., 2021), mental health issues are the densest issues in IOP studies during COVID-19 pandemic. Mental health has been an emerging issue since pandemics caused a significant increase in the problem (International Labour Organization, 2021).

Six clusters of themes formed from the cluster density analysis, namely mental health, health care worker as a research subject, specific workplace issues, digital technology issues, methodologies used and country. In every cluster, we provide an in-depth look into the issues ranging from scope, causes, risk factors and implications.

#### Mental health

The prominent theme from the bibliometric analysis in IOP research is mental health. This indicates the dominance of the emergence of the keywords related to this theme, which is consistent with the result at the beginning of the COVID-19 pandemic (Zambrano et al., 2021). The effect of the COVID-19 pandemic on mental health has received considerable attention because at least 10% of the population reported experiencing mental health problems (Gloster et al., 2020). The population experienced an increase in several mental health problems during the COVID-19 pandemic, ranging from personal symptoms to symptoms related to performance and the environment, such as doubting knowledge and skills and feeling uncomfortable within the team (Vanhaecht et al., 2021). The prominent mental health problems experienced during the COVID-19 pandemic are acute stress disorder, anxiety and depression, with chest pain, physical exhaustion and sleep disturbance as common symptoms (Van Roekel, Van Der Fels, Bakker, & Tummers, 2021; Wang et al., 2021; Yang et al., 2021). There are several risk factors identified: (1) social connectivity, including social support (Gloster et al., 2020; Ye et al., 2020) and lower-level communication with friends (Tahara, Mashizume, & Takahashi, 2020); (2) demographic factors, including education level (Gloster et al., 2020), female gender (Prati, 2021) and employment status (Nam, Eum, Huh, Jung, & Choi, 2021); (3) personal factors, including self-compassion (Kotera, Mayer, & Vanderheiden, 2021), personality traits and coping (Cook, Hassem, Laher, Variava, & Schutte, 2021; Osimo, Aiello, Gentili, Ionta, & Cecchetto, 2021; Smith et al., 2021), resilience (Cook et al., 2021), work satisfaction (Tahara et al., 2020) and work engagement (Kotera et al., 2021); (4) environment and work factors, including high-risk working area (Ruiz-Fernández et al., 2020), work stressor (Hu, Dai, Wang, Zhang, Li, & He, 2021), work responsibility (Parthasarathy, Jaisoorya, Thennarasu, & Murthy, 2021), work hour (Britt et al., 2020) and organisational support (Cook et al., 2021). These mental health problems will interfere with long-term health conditions (Zara, Settanni, Zuffranieri, Veggi, & Castelli, 2021) and work functioning (Fu, Greco, Lennard, & Dimotakis, 2021).

#### Health care worker as a research subject

Workplace research during the COVID-19 pandemic focused more on the area of health-related businesses. However, some researchers have also examined business areas affected by the pandemic, such as retail, hospitality, tourism (Aburumman, 2020; Childs, Gokcecik, Yoon, & Lee, 2021), manufacturing (Belhadi et al., 2021; Görlich & Stadelmann, 2020) and sport (Kennedy & Kennedy, 2020; Parnell, Bond, Widdop, & Cockayne, 2021). Following the healthcare business as focus, research participants were mostly health care workers, both nurses and physicians. Consistent with the impact of the previous outbreak, health care workers were the most vulnerable group to mental health problems (Shah et al., 2020), and they have a higher risk of experiencing long-term mental health effects (Nelson & Lee-Winn, 2020). Health care workers’ mental health problems increased because of irregular working hours, higher levels of exposure to illness, fear of infection and lack of adequate PPE, amongst others (World Health Organization, 2021; Yang et al., 2021). Anxiety and depression were mental health problems frequently experienced, with insomnia, sleeping disorders and burnout as risk factors (Chen et al., 2021; World Health Organization, 2021), and these are often reported by workers who work with COVID-19 patients (Parchani et al., 2021; Vagni, Maiorano, Giostra, & Pajardi, 2020; Van Roekel et al., 2021). During this pandemic, traumatic stress in health care workers was also related to moral injury, because they were often facing difficult conditions in making decisions (Litam & Balkin, 2021), especially those with clinical responsibility (Parthasarathy et al., 2021). Regarding the conditions, providing mental health programmes for health care workers is urgently needed, both as preventive and curative action (Crittenden, Spieker, & Landini, 2021; Yang et al., 2021). Besides developing internal ability programmes such as increasing self-efficacy (Vagni et al., 2020), resiliency (Kelly, Uys, Bezuidenhout, Mullane, & Bristol, 2021), effective appraisal and coping (Cook et al., 2021; Ji, Han, Deng, & Lu, 2021; Pearman, Hughes, Smith, & Neupert, 2020), it is essential to provide social support (Husted & Dalton, 2021; Ji et al., 2021) and health service such as telecounselling that may feasibly improve mental health conditions (Gupta et al., 2021).

#### Specific workplace issues

Based on dominant keywords, specific workplace issues can be categorised into two categories. Firstly, workplace mental health consists of mental health problems and related variables, namely burnout, occupational stress, fatigue and workload. Secondly, general workplace changes because of the COVID-19 pandemic covered job insecurity, work from home (WFH) or remote work, leadership, job satisfaction, work engagement, work or task performance, entrepreneurship, work–life balance and productivity. In the context of this pandemic situation, mental health issues as the first category are related and centred on the issue of burnout. Most workers experience burnout during pandemics (Denning et al., 2021) with predictors such as demographic, personal health problems, work environment and personal factors. Demographic factors include gender, parental status, marriage status (Duarte et al., 2020), educational level (Shoja et al., 2020) and age (Littzen, 2021). Personal health problems consist of insomnia, somatic symptoms (Rodríguez-López, Rubio-Valdehita, & Díaz-Ramiro, 2021), stress and depression (Duarte et al., 2020) that were confirmed or suspected with COVID-19 infection (Sarboozi Hoseinabadi, Kakhki, Teimori, & Nayyeri, 2020). Work environment factors are work change (task, setting and team) (Rodríguez-López et al., 2021; Sklar, Ehrhart, & Aarons, 2021), COVID-19 exposure at work (Sarboozi Hoseinabadi et al., 2020), salary reduction (Duarte et al., 2020), hospital resources (Sarboozi Hoseinabadi et al., 2020), work overload (Rodríguez-López et al., 2021; Shoja et al., 2020), the shift of work (Shoja et al., 2020), period of working hours (Dimitriu et al., 2020) and workload (Dimitriu et al., 2020). Personal factors include negative coping strategies (Crescenzo et al., 2021), risk concerns (Sousa, Almeida, & Leal, 2021), job expectations (Rodríguez-López et al., 2021), job stress (Sarboozi Hoseinabadi et al., 2020), lower job dedication (Huang et al., 2021), emotional intelligence (Olatunji, Idemudia, & Owoseni, 2020), life satisfaction and resilience (Di Trani, Mariani, Ferri, De Berardinis, & Frigo, 2021). Most of the studies were conducted on healthcare workers, whilst others provide a different point of view by examining burnout in participants from fashion retailing workers (Rodríguez-López et al., 2021) and social workers (Garcia, 2021). Concerning the cases, several suggestions arise to prevent and minimise the burnout effect, such as by building positive attitude and social support strategies (Crescenzo et al., 2021), utilising boundaries at work (Rapp, Hughey, & Kreiner, 2021), decreasing workload, promoting procedural justice and professional identification (Correia & Almeida, 2020).

Meanwhile, most studies focused on problems, and the other research provided alternative solutions to dealing with workplace mental health problems. The first solution is a personal-oriented solution that works on individuals psychologically and physically. Psychologically, research shows that strengthening hope and optimism is one of the keys in dealing with coronavirus-related anxiety problems (Prazeres et al., 2020). Physically, individuals are encouraged to remain active to overcome work fatigue, because a sedentary lifestyle (as predominantly practised during the pandemic) increases worker fatigue (Koohsari et al., 2021). The second solution is a social-oriented solution that works on building social support. Tahara et al. (2020) describe that participants who experienced poorer mental health conditions during this pandemic tend to seek social support. The pandemic as a stressful condition may drive individuals to reactivate dormant ties as a coping mechanism (Yang, Soltis, Ross, & Labianca, 2021). The third solution is a system-oriented solution that focuses on policy and systems in the workplace. Modification to the work environment is important to minimise mental health problems and burnout. Tan et al. (2020) suggest that organisations provide adequate training, avoid long shifts of more than 8 h and promote safe work environments, especially for health care companies. Modification in work design, suggested by Sklar et al. (2021), implies that organisations should limit task, setting and team-related work changes to the extent possible. Emotional support in the work environment also needs to be fostered by implementing the stepped care model (Price, Becker-Haimes, & Benjamin Wolk, 2021).

The second category implies general workplace changes during the pandemic, ranging from worker level to policies. At the worker level, millions of jobs were lost because of the pandemic (International Labour Organization, 2020). It not only impacted job insecurity but also affected emotional and mental health conditions (Lin, Shao, Li, Guo, & Zhan, 2021; Obrenovic, Du, Godinic, Baslom, & Tsoy, 2021), job performance (De Angelis, Mazzetti, & Guglielmi, 2021) and well-being (Stankevičiūtė, Sanchez-Hernandez, & Staniškienė, 2021). At the policy level, immediate action at the beginning of the pandemic was related to preventing the COVID-19 from spreading by applying for WFH, which has brought a different way of working (Hartner-Tiefenthaler, Goisauf, Gerdenitsch, & Koeszegi, 2021). Interestingly, besides side effects like worsening presenteeism (Shimura, Yokoi, Ishibashi, Akatsuka, & Inoue, 2021) and increasing emotional exhaustion (Chong, Huang, & Chang, 2020), workers perceived more productivity during WFH (Zappalà, Toscano, & Topa, 2021), and it decreased psychological and physical stress responses (Shimura et al., 2021). Some research highlights that the effectiveness of WFH depends on leadership (Shockley, Allen, Dodd, & Waiwood, 2021) and managerial control policies (Hartner-Tiefenthaler et al., 2021; Irshad et al., 2021).

#### Digital technology issues

The pandemic has accelerated digitalisation and automation in various business areas, either in technologies that relate directly to the virus treatment or in areas that assist people to adjust to the crisis conditions (Brem, Viardot, & Nylund, 2021). It is dominated by telemedicine in health care (Rivest, Caron, & Desbeaumes Jodoin, 2021), e-learning in education (Alqahtani & Rajkhan, 2020) and teleworking in almost any kind of business (Zhang, Yu, & Marin, 2021). The absorption of telemedicine in the first year of the pandemic increased by 50% compared to the same period in the previous year (Koonin et al., 2020) and provided a variety of services from physical to mental health problems (Thippaiah, Harbishettar, Kumar, & Pandurangi, 2020; Watts et al., 2020). A meta-analysis showed that telemedicine is effective for some mental health interventions and reduce client waiting time compared to traditional therapy (Bennett, Ruggero, Sever, & Yanouri, 2020). In the workplace context, during the pandemic, the employee assistance programme (EAP) transformed into telehealth because it was primarily delivered virtually (Couser, Nation, & Hyde, 2021). Nevertheless, virtual EAP or telehealth still faces a low adoption rate for many reasons, but eventually many clients become more familiar with the new modes of delivery (Couser et al., 2021).

#### Methodologies used

The keyword frequently appearing in research methods is ‘cross-sectional study’. As one of the most widely used designs in applied psychology research (Austin, Scherbaum, & Mahlman, 2004), the cross-sectional study is particularly suitable for estimating the prevalence of behaviour or disease in a population (Sedgwick, 2014). The advantages of this design include simplicity, data acquisition speed, cost-effectiveness, short data collection period and minimal participant burden (Sedgwick, 2014; Taris, Kessler, & Kelloway, 2021). This design has an eminent advantage for data collection in COVID-19 research because most participants have direct exposure to COVID-19 handling. Several researchers compare different types of workgroups, including those based on demographics (Duarte et al., 2020; Tahara et al., 2020), position and responsibilities (Denning et al., 2021; Sarboozi Hoseinabadi et al., 2020) and personal factors (Tahara et al., 2020). Therefore, the researchers used cross-sectional designs because they are well-suited for testing assumptions about the relationships of interest and provide a clear impression of the state of affairs in an organisation or amongst a group of workers at a given point in time (Taris et al., 2021). The other keywords that emerged in this cluster are ‘survey’ and ‘questionnaire’. The methods are frequently used during the COVID-19 pandemic because of the ease of collecting extensive data in a faster way and the possibility to continue online collection during the restriction period (Singh & Sagar, 2021). However, Singh and Sagar (2021) raised several concerns about the methods that could impact the scientific quality of survey findings.

#### Country

Several countries that appear as the dominant keywords in COVID-19-related workplace research include the USA and China. Both countries had the worst outbreak records at different times and contribute considerably to COVID-19 research (Wang & Tian, 2021; Zhai et al., 2020). By 2021, the USA’s research contributed 21.3% of the total world research and focused on intervention and vaccines, whilst China has reached 20.7% that emphasised clinical features, virology and immunology, epidemiology, detection and diagnosis (Wang & Tian, 2021). As the top two COVID-19 research contributors, China showed notable works in the early pandemic, especially in clinical features and complications, such as Huang et al.’s (2020) work, since the first case was found in Wuhan, but by July 2021 China was overtaken by the USA (Wang & Tian, 2021).

### Practical implications

The study findings provide an overview of the current prominent issues during the pandemic and potential areas for further research in IOP literature. Firstly, this study provides an in-depth overview of the salient issues, ranging from causes, implications and other variables involved. Practitioners can use these findings as a basis for policymaking. As an example, in mental health issues, studies found several protective factors that can be taken into consideration when making policies or interventions. Secondly, the bibliometric analysis found that the most-discussed issues are mental health, stress and burnout, categorised as worker-level issues. Besides the main issues, many studies also examined moderating factors like demographics, personal factors and organisational environmental factors. Meanwhile, although discussions related to organisation-level issues, such as WFH (Chong et al., 2020; George, Atwater, Maneethai, & Madera, 2021; Min, Peng, Shoss, & Yang, 2021), leadership (Hu, He, & Zhou, 2020; Newman & Ford, 2021; Sergent & Stajkovic, 2020) and human resources (HR) systems (Bierema, 2020; Risley, 2020), were presented in the study, the number and coverage is still limited. This result indicates that study and discussion about work practice and system were potentially because the COVID-19 pandemic affected behavioural changes in various sectors (Cucchiarini, Caravona, Macchi, Perlino, & Viale, 2021; Sheth, 2020), and hence an adaptation system is urgently needed. Thirdly, more than 70% of articles focused on the health care business area. However, some business areas were seriously hit by the pandemic; for example, airlines experienced about 60% decline and the manufacturing industry should modify their operations. It implies that further research may focus on broadening the business areas such as tourism industry, manufacturing industry and information technology industry. Fourthly, the cross-sectional method provides an overview of the workplace during the pandemic; however, several problems have a long-term impact. Hence, longitudinal studies may be beneficial in investigating the problem.

## Limitation and recommendations

Two study limitations should be considered when evaluating and interpreting the findings of this study. Firstly, one of the inclusion criteria in this study was English-based articles; furthermore, studies with non-English were excluded from the analysis. Secondly, this study used articles from a fairly long period of publication, whereas the COVID-19 crisis shows a changing pattern, and therefore details focusing on specific phases in the crisis are less likely to be captured.

## Conclusion

Current studies in the IOP field during the COVID-19 pandemic focused on mental health and worker-level issues. The results imply that most studies work on an individual level. Therefore, future research on the group and organisational level is needed to give a comprehensive picture of the impact of the COVID-19 pandemic in the workplace, such as adaptation of HR systems and implementation of organisational development. It will be beneficial as the basis to build systems and policies during and after the pandemic in the workplace. Previous studies focused more on the health care business and workers. Therefore, studies in broader areas are still needed as other business areas also experienced the indirect impact of the COVID-19 pandemic.
